# Supplementary material for: Urine NMR Metabolomics for Precision Oncology in Colorectal Cancer
Source: Int J Mol Sci. 2022 Sep 22;23(19):11171. doi: 10.3390/ijms231911171 (PMC9569997; doi:10.3390/ijms231911171)
Supplement: Supplementary file 1 [file ijms-23-11171-s001.zip › NMR_metabolomics_CRC_SUPPL_vSubmitted.pdf]

Urine NMR metabolomics for precision oncology in colorectal cancer

Jesús Brezmes[1,2], Maria Llambrich[1,2], Raquel Cumeras[2,3,1*] and Josep Gumà[3]

Supplementary Figures

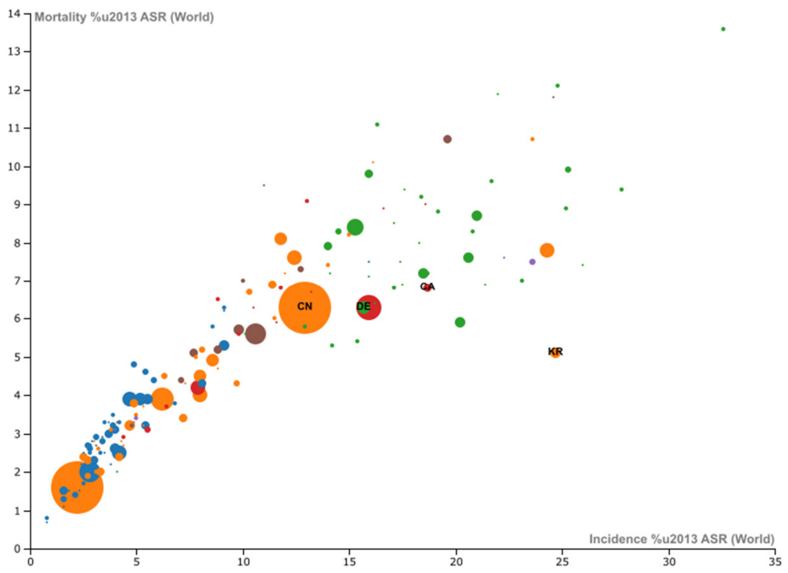

Figure S1: *Incidence versus mortality data from countries worldwide, with names of the countries of the studies from which this review is based on. The image is based on data from GloboCAN2018. The circle size is proportional to the country's population. The color indicates the continent: blue Africa, orange Asia, green Europe, red North America, purple Australia, brown South America and Central America. Countries highlighted are the ones included in the study: CA: Canada; CN: China; DE: Germany; KR: Korea Republic of (South Korea).*

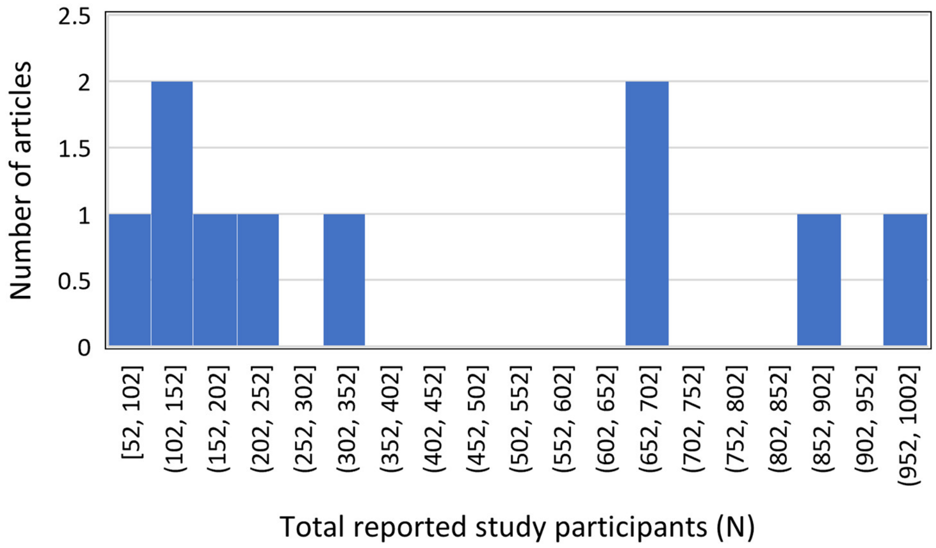

Figure S2: *Histogram of the total reported number of participants (N) per study included in the articles selected for the systematic review.*
